# Task-sharing with artificial intelligence: a design hypothesis for an Emergency Unit in sub-Saharan Africa

**DOI:** 10.11604/pamj.2021.38.387.20557

**Published:** 2021-04-20

**Authors:** Camillo Lamanna

**Affiliations:** 1 University of New South Wales, Kirby Institute, Wallace Wurth Building, Kensington, New South Wales 2052, Sydney, Australia

**Keywords:** Artificial intelligence, emergency unit design, task-sharing

## Abstract

In sub-Saharan Africa, there is a significant unmet need for emergency care, with a shortage of trained providers. One model to increase the number of providers is to task-share: roles traditionally filled by clinicians are shared with lay workers who have received task-specific training. Separately, there has been much recent interest in the possible implications of artificial intelligence (AI) on healthcare. This paper proposes that, by combining the task-sharing model with AI, it is possible to design an Emergency Unit (EU) that shares the tasks currently undertaken by physicians and nurses with lay providers, with the activities of lay providers guided and supervised by AI. The proposed model would free emergency care clinicians to focus on higher-acuity and complex cases while AI-supervised routine care is provided by lay providers. The paper outlines the model for such an implementation and considers the potential benefits to patient care, as well as considering the risks, costs, effect on providers, and ethical questions. The paper concludes that AI and healthcare workers can operate as a team, with significant potential to augment human resources for health in sub-Saharan Africa.

## Essay

In sub-Saharan Africa, there is a significant unmet need for emergency care [1]. Multiple barriers to timely and effective care have been identified, ranging from gaps in pre-hospital care networks to infrastructural limitations [2,3]. These barriers are often compounded by a lack of providers who are trained in emergency care. Many hospitals in the region do not have a dedicated Emergency Unit (EU); most have no physicians or nurses who have received specialist training in emergency care [4]. Moreover, both physicians and nurses are expensive to train and to retain, and the WHO predicts that the shortage of African healthcare workers will worsen over the coming decade [5]. Against this backdrop, innovative solutions could deliver significant gains to emergency care in the region. One proposed model to increase the number of healthcare providers is to “task-share” roles traditionally filled by highly- (but broadly-) educated physicians and nurses with healthcare workers who have received (brief and relatively inexpensive) task-specific training. From managing hypertension to HIV, and from recording vital signs to performing emergency surgery, there is evidence from sub-Saharan Africa that task-sharing can help address the gap in human resources for health [6-10]. Separately, there has been much recent interest in the possible implications of artificial intelligence (AI) on healthcare. There are multiple instances in fields including dermatology [11], oncology [12], and radiology [13], where trained AIs have matched or outperformed experienced clinicians in diagnosis. While the lay press subsequently concludes that AI may come to replace physicians, the academic literature tends to be more circumspect. Especially in the field of emergency medicine, AI is predicted to function mainly as a decision aid, which will assist healthcare providers in making more timely and accurate decisions [14, 15]. This paper claims that, by combining the task-sharing model with AI, it would be possible in principle to design an Emergency Unit (EU) that operates safely and effectively, while making substantial cost savings relative to the traditional model of the EU. More specifically, the multiple and wide-ranging tasks currently undertaken by physicians and nurses could be shared with lay providers (LPs), who have been trained to undertake specific tasks, with the overall flow and management of most low- and moderate-acuity patients supervised by AI. This paper suggests that, rather than obviating the need for physicians and nurses, the proposed model would free them to focus on higher-acuity and complex cases which require a broader range of skills, while the AI-supervised task of offering high-quality but relatively routine care is shared with LPs.

**Design hypothesis for AI-supervised low- and moderate-acuity care:** this section outlines a model of AI-supervised care in the EU. It argues that a team of four LPs, supervised by AI, can safely deliver high-quality patient care in an efficient, cost-effective manner. Practically, AI ‘supervision’ will involve the AI system giving LPs instructions and prompts (via an electronic device) to direct patient care. The inputs that AI will receive include: responses to AI-directed questions, vital signs and basic examination findings, results of bedside, imaging and/or laboratory tests. The proposed patient journey begins with a dedicated triage LP who takes the patient´s demographic information, history, and performs a focused examination. AI-assisted prompts will ensure that relevant questions are covered, tailored to the presenting complaint. AI interprets the history in conjunction with the clinical findings and arranges the appropriate further investigations and treatment. The patient then moves to a procedural LP who performs routine EU procedures (e.g., ECG recording, cannulation/venepuncture) as necessary, directed by AI. Next, the patient moves to a treating LP who can administer medications, as directed by the AI system, and can perform basic orthopaedic procedures (e.g., apply plaster casts). The patient then moves to a waiting area where a nursing LP re-checks vital signs and engages in shared decision-making with the patient as directed by AI-given prompts. The ultimate disposition of the patient, guided by the interpretation of the history, of clinical examination, of investigations, is decided by this final shared decision-making process, overseen by the AI system. Finally, the patient is provided with discharge and follow-up instructions, or a handover is generated to clinical staff if the patient is being admitted or transferred. Importantly, it should be noted that the AI-supervised model can be operated in parallel; namely, multiple teams of four LPs can operate side-by-side simultaneously. An example of AI-supervised patient flow is given in Table 1.

**Table 1:** artificial intelligence-supervised task-sharing example for a patient with cough and fever

	Lay provider role	Artificial intelligence role
**Triage**	Takes patient history	Directs history and examination
	Performs brief examination including vital signs	Interprets history, examination and vital signs
		Documents interaction on electronic health record
		electronic health record
		Requests blood tests and chest X-ray
**Procedures**	Performs venepuncture	Interprets chest X-ray
		Interprets blood results
		Prescribes immediate treatment
**Treatment**	Gives anti-pyretic	
**Nursing**	Rechecks vital signs	Diagnoses community-acquired pneumonia
	Explains likely diagnosis, treatment and expected prognosis with follow-up advice	Gives shared decision-making template to lay provider
		Prescribes course of antibiotics as per hospital guidelines

**Impact on patient care:** almost two-thirds of patients presenting to African EUs require assessment that is either routine or moderately urgent [16]. Evidence-based guidelines exist for most of these presentations. Therefore, an AI-supervised team that follows clinical guidelines would likely be able to manage most patients presenting to EUs in sub-Saharan Africa, based on the case-mix of EU presentations [17, 18]. Regulatory bodies typically assert that standardised, evidence-based, guideline-driven practice leads to improved patient care. Despite this, emergency care practitioners (ECPs) frequently do not adhere to guideline recommendations [19, 20]. One study showed that AI-driven care was 39% more consistent with evidence-based guidelines than physicians´ practice [21]. There is reason, therefore, to believe that an AI-supervised EU would also deliver consistent, high-value care. Patient care would also be less vulnerable to human error. Medical error is thought to be a significant cause of morbidity and mortality - it has been claimed to be the third-leading cause of preventable death in the US [22]; though data from Africa are scarce, the available evidence suggests errors are no less prevalent [23]. In the EU setting in particular, a significant proportion of errors are due to drug prescription errors [24]. AI-supervised prescriptions will likely reduce error, as doses, drug interactions, and allergies can all be checked electronically [25]. A second significant source of error is misinterpretation of bedside, radiological, and laboratory findings [26, 27]. Researchers have recently demonstrated that machine learning algorithms can interpret chest X-rays and computed tomography (CT) heads at a level comparable to radiologists [28, 29]. There is good reason to believe that similar tools will soon be able to interpret laboratory findings with greater accuracy than general physicians [30], and interpret significant ECG abnormalities at a level comparable to a cardiologist [31]. Therefore, an appropriately trained AI system will foreseeably improve patient safety due to increased accuracy of diagnostic test interpretation.

**Risks:** as the proposed model is hypothetical, there are no data comparing clinician-led to AI-led emergency care. A recent observational study in India found that Watson for Oncology, an AI-driven cognitive computing system, made treatment recommendations that were 93% concordant with a multidisciplinary team of oncology experts [32]. This demonstrates the feasibility of using AI-powered decision aids safely in low- and middle-income countries. However, all decision-making that utilises machine learning is vulnerable to two categories of error: first, that injudicious use of machine learning algorithms can lead to overconfidence in inaccurate predictions (a training problem); second, that algorithms replicate human errors and reflect existing systematic biases in the data (a training set problem) [33]. These risks can be can only be mitigated by thoughtful construction based on data that reflects the patient population and is scrupulously examined for bias and error by researchers in tandem with developers. Furthermore, as it is crucial that the AI system have a low threshold for involving an experienced ECP in cases of uncertainty, perhaps the proposed system would not in fact reduce ECP workload. This concern has been voiced for Watson for Oncology [34]. However, machine learning algorithms can learn from mistakes and misclassifications and this can be harnessed to create a self-improving system. Finally, the safety of AI-led care also depends on the accuracy of the inputs with which it is provided, and the adherence of LPs to its recommendations. Evidence suggests that lay health workers comply well with electronic prompts (which would be used to guide management in the proposed implementation) [35]. Nonetheless, the risk of non-adherence to the AI-led treatment recommendations needs to be mitigated.

**Impact on providers:** while the evidence suggests that LPs can be as effective as traditionally-trained healthcare workers [36], it is often reported that LPs find the role unsatisfying, due to low compensation, lack of supervision and limited career progression [37, 38]. Thus, the need to provide feedback and mentorship for LPs, as well as a clearly-defined career trajectory, is foreseen in order to maintain sustainable, high standards of care [39]. Similarly, the effect on ECPs´ training and practice needs to be considered. On the one hand, an LP team will free resources for physicians and nurses to manage higher-acuity and more complex cases. Anecdotally at least, ECPs tend to prefer managing high-acuity cases. This would likely increase the acceptability of the proposed model to ECPs. On the other hand, ECPs may feel that LPs are seeing the ‘bread and butter´ of EU presentations. This could be perceived both as an encroachment on the role of the ECP and as potentially dangerously de-skilling: for example, if an emergency physician no longer sees routine asthma presentations, (s)he may be less able to manage life-threatening asthma in the EU. Hence, the integration of the model needs to be carefully considered to contribute positively to the education and workload of ECPs.

**Cost:** based on existing data for personnel costs and emergency physician productivity [40, 41], one could expect an AI-supervised team of LPs to save over 80% of the wage bill relative to the traditional model. Moreover, there will likely be savings beyond labour costs. It is well-documented that healthcare workers frequently order unnecessary investigations and provide non-evidence-based or ‘low-value´ treatment [42, 43]. These have significant associated financial costs, as well as exposing patients to unnecessary risks. An AI-based model, which only orders investigations and treatments as per evidence-based guidelines and algorithms would, in principle, not incur these costs. Therefore, one should expect the running costs of an AI-supervised EU to be lower still. On the other hand, a project that involves developing a robust AI system is likely to be significant. It is worth making two observations: firstly, the AI system only needs to be developed once, therefore this (unlike training physicians and nurses) is not a recurring cost; secondly, the market for such a product would be substantial - in Africa, there are an estimated 5,000 public hospitals, however the model could feasibly be employed in any EU [44].

**Ethics:** mistakes will be made in an AI-supervised EU. Some of these mistakes will lead to ‘AI-atrogenic’ morbidity and mortality. The question of whether the mortality benefits outweigh the harms is an empirical one, but the question of how to design and test such a system is ethical. AI-led care can be delivered in areas where experienced ECPs are in short supply. Should an AI-supervised EU be compared to existing care? Or to a traditional EU run by ECPs? Or to an EU run by ECPs and with AI-directed care (and no lay providers)? Given the complex and problematic history of medical research in Africa [45], it is crucial that the development and implementation of an AI-led system have substantial, if not overall, direction from African stakeholders, and full understanding and buy-in from communities. Earlier this year, Google completed building its first African AI hub in Ghana, recognising the technological talent and future opportunities for AI on the continent [46].

## Conclusion

Africa has a recent history of ‘leap-frogging’ its Western cousins in the adoption of technology; recent examples include drone-delivery services for medical products in Rwanda and bank accounts held via mobile phones in East Africa. Emergency care could become another example whereby a limitation (a paucity of trained ECPs) forces an innovation (AI-led emergency care) which surpasses the equivalent in the West. This essay has proposed a model whereby a team of LPs, supervised by AI, provides routine care for low- and moderate-acuity patients in EUs in sub-Saharan Africa. It has been argued that, if correctly implemented, such a model could deliver consistent high-quality patient care and widen access with substantial running cost savings relative to the traditional model of EU care. Considering possible risks, effects on providers, and ethics, it was concluded that while AI-supervised care may offer benefits, the design, testing and implementation would need to be judiciously and carefully guided by a range of African stakeholders. The role of AI in facilitating healthcare delivery resource-poor settings is increasingly being realised. The hypothesised model could become part of a solution to enrich human resources for health in Africa.
